# *ret*/PTC-1 expression alters the immunoprofile of thyroid follicular cells

**DOI:** 10.1186/1476-4598-7-44

**Published:** 2008-05-27

**Authors:** Karen Denning, Paul Smyth, Susanne Cahill, Jinghuan Li, Richard Flavin, Sinead Aherne, John J O' Leary, Orla Sheils

**Affiliations:** 1 Department of Histopathology, University of Dublin, Trinity College, Dublin, Ireland; 2 Department of Histopathology, Institute of Molecular Medicine, Trinity Centre for Health Sciences, St James Hospital, Dublin 8, Ireland

## Abstract

**Background:**

Hashimoto Thyroiditis (H.T.) is a destructive autoimmune thyroid condition whose precise molecular pathogenesis remains unclear. *ret*/PTC-1 is a chimeric transcript which has been described in autoimmune thyroid disease (AITD) and thyroid neoplasia. The purpose of this study was to observe the immunogenic effect exposure to H.T. and control lymphocyte supernatant would have on normal (Nthy-ori) and *ret*/PTC-1 (TPC-1) expressing thyroid cell line models.

**Results:**

A 2 × 2 matrix comprising Nthy-ori and TPC-1 cell lines and H.T. and control lymphocyte supernatant was designed and utilised as follows; activated lymphocytic supernatant from a H.T. and normal control were co-cultured with a cell line derived from normal thyroid (Nthy-ori) and also a cell line derived from a papillary thyroid carcinoma that endogenously expresses *ret*/PTC-1 (TPC-1). The co-cultures were harvested at 0, 6 and 18 hour time points. Gene expression analysis was performed on RNA extracted from thyrocytes using TaqMan^® ^Immune profiling Low-Density Arrays (Applied Biosystems, CA, USA) comprising gene expression markers for 93 immune related targets plus 3 endogenous controls.

Stimulation of the normal thyroid cell line model with activated T cell supernatant from the H.T. donor yielded global up-regulation of immune targets when compared with control supernatant stimulation. In particular, a cohort of targets (granzyme B, CD3, CD25, CD152, CD45) associated with cytotoxic cell death; T cell receptor (TCR) and T cell signaling were up-regulated in the normal cell line model. When the *ret*/PTC-1 expressing thyroid cell line was co-cultured with H.T. lymphocyte supernatant, in comparison to control supernatant stimulation, down-regulation of the same subset of immune targets was seen.

**Conclusion:**

Co-culturing H.T. lymphocyte supernatant with a normal thyroid cell line model leads to over-expression of a subset of targets which could contribute to the pathogenesis of H.T. via cytotoxic cell death and TCR signalling. Stimulation of the *ret*/PTC-1 positive cell line with the same stimulus led to a down-regulated shift in the gene expression pattern of the cohort of immune targets. We hypothesize that *ret*/PTC-1 activation may dampen immunogenic responses in the thyroid, which could possibly facilitate papillary thyroid carcinoma development.

## Background

Lymphocytic infiltration is a feature of many thyroid diseases, both benign and malignant. Hashimoto Thyroiditis (H.T.), an autoimmune thyroid disease, is characterised morphologically by inflammatory infiltrate and diffuse fibrosis which typically leads to progressive thyrocyte depletion. This loss of thyrocyte capacity results in impaired thyroid hormone production and clinical hypothyroidism. However the exact molecular mechanisms by which thyrocyte destruction occurs remains to be seen.

The *ret*/PTC oncogenes represent activated, rearranged forms of the *RET *proto-oncogene. They were initially thought to be specific for papillary thyroid carcinoma (PTC), but have subsequently been found in benign thyroid conditions such as Hashimoto Thyroiditis [1-3]. The *ret*/PTC oncogenes are formed as a result of chromosomal inversions within chromosome 10 resulting in the fusion of the tyrosine kinase domain of *RET *to another donor gene. The most commonly detected rearrangement is *ret*/PTC-1 (~70%), in which the tyrosine kinase moiety of *c-ret *is fused to the 5' end of the H4 gene [4].

The observation that *ret*/PTC-1 was not restricted to PTC raised the possibility that a) H.T. might represent an early malignant state to which some patients are able to mount an immune response, or b) that *ret*/PTC-1 occurs in the absence of malignancy and is not specific for PTC.

In either event *ret*/PTC-1 activation is associated with florid lymphocytic infiltration. The objective of this study was to investigate the immune response elicited by a normal and endogenously expressing *ret*/PTC-1 thyroid cell line after co-culture with autoimmune H.T. and normal lymphocytic supernatant using functional genomics. Immunoprofiling TaqMan^® ^low density arrays were used to evaluate the expression profiles of *ret*/PTC-1 positive and negative thyrocytes over a time course of co-culture with activated T cell supernatant.

## Results

TaqMan^® ^low density array immunoprofiling data was analyzed using SDS 2.1 software (Applied Biosystems, CA, USA). Independent RQ studies were carried out for each cell line. In both cell lines (Nthy-ori and TPC-1), the 6 hour time point displayed peak expression differentials compared with T_0 _and between different stimuli (Normal and H.T.).

### Nthy-ori co-culture gene expression patterns

**Figure 1:**
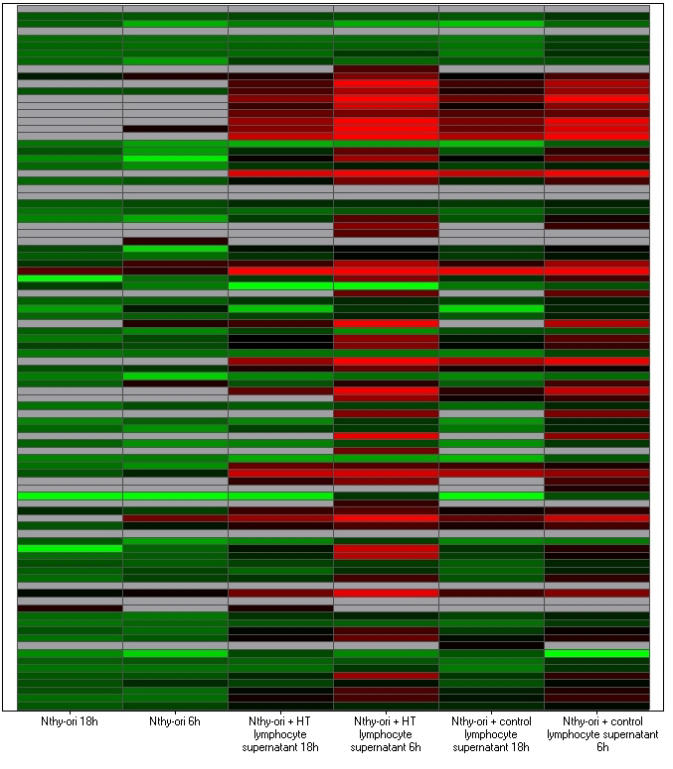
Heat map of Nthy-ori co-culture. This heat map represents all 93 immune targets assayed (plus endogenous control) at each time point/co-culture combination that RNA was extracted at. Relative quantification (RQ) values for each target were used to create the heat map. Red denotes genes with relative increased expression while green denotes genes with relative decreased expression.

In the normal thyroid cell line model (Nthy-ori), exposure to H.T. lymphocyte supernatant led to global up-regulation of 92 inflammation targets (with the exception of one, Heme oxygenase-1 where expression between each cohort was equivocal) in comparison to stimulation with normal control lymphocytes. The Nthy-ori co-culture heat map (figure 1) illustrates expression of the 93 immune related targets assayed plus endogenous control at each co-culture combination and time point analyzed. The heat map demonstrates increased expression levels of targets exposed to H.T. lymphocyte supernatant compared to control at the 6 hour time point. It also demonstrates a reduced level of gene expression in Nthy-ori cells that were not exposed to stimulus at the 18 and 6 hour time points.

**Figure 2:**
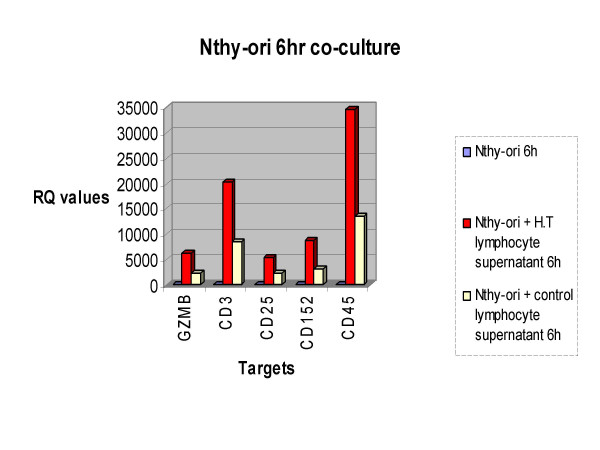
Nthy-ori 6 hour co-culture. Figure 2 displays targets that were up-regulated in *ret*/PTC-1 negative thyrocytes when co-cultured with Hashimoto Thyroiditis lymphocyte supernatant in comparison to exposure to control lymphocytic supernatant after a 6 hour interval.

Figure 2 displays a subset of targets which were over-expressed in the normal thyroid cell line model (Nthy-ori) when exposed to H.T. lymphocytic supernatant compared to control at the 6 hour time point. They included granzyme B (GZMB), a known effector in cytotoxic cell death, CD3, CD25, CD152 and CD45 all of which are involved with the T cell receptor (TCR) or signaling in the T cell.

**Figure 3:**
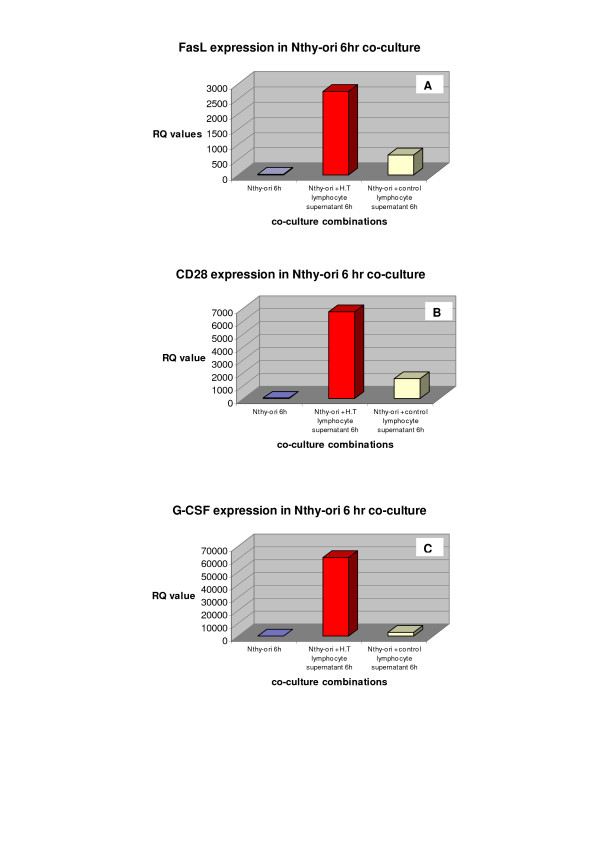
Expression levels for FasL, CD28 and G-CSF. Figure 3a-c displays gene expression levels for FasL(3a), CD28(3b) and G-CSF(3c) expression in the Nthy-ori 6 hour co-culture. Each target is over-expressed in the normal thyroid cell line when exposed to Hashimoto Thyroiditis lymphocyte supernatant in comparison to exposure to control lymphocyte supernatant.

Figure 3a–c displays the gene expression level of Fas ligand (FasL), CD28 and granulocyte colony stimulating factor (G-CSF) in the Nthy-ori co-culture. These targets also displayed increased expression levels in the normal thyroid cell line model which was exposed to H.T. lymphocyte supernatant when compared to exposure to control supernatant.

### TPC-1 co-culture gene expression patterns

**Figure 4:**
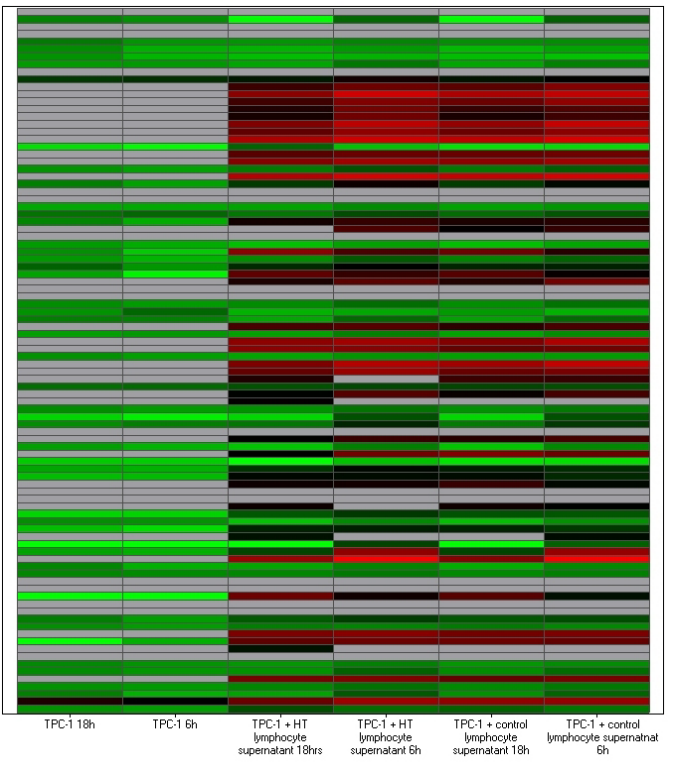
Heat map of TPC-1 co-culture. This heat map represents all 93 immune targets assayed (plus endogenous control) at each time point/co-culture combination that RNA was extracted at. Relative quantification (RQ) values for each target were used to create the heat map. Red denotes genes with relative increased expression while green denotes genes with relative decreased expression.

In the *ret*/PTC-1 harboring cell line, approximately 40% of targets (with a relative quantification (RQ) value >100) were down-regulated when stimulated with H.T. lymphocyte supernatant in comparison to exposure with control supernatant. The TPC-1 co-culture heat map (figure 4) illustrates the reduction in gene expression at the TPC-1 18 and 6 hour time points where there is no exposure to supernatant.

**Figure 5:**
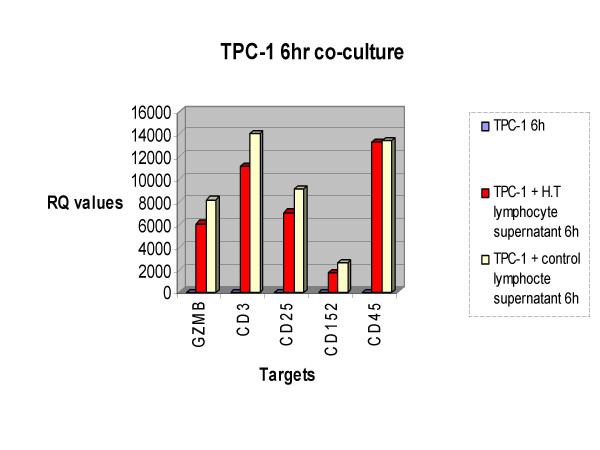
TPC-1 6 hour co-culture. Figure 5 displays how targets that were up-regulated in *ret*/PTC-1 negative thyrocytes (figure 2) are down-regulated in *ret*/PTC-1 positive thyrocytes when co-cultured with Hashimoto Thyroiditis lymphocyte supernatant in comparison to exposure to control lymphocytic supernatant after a 6 hour interval.

Figure 5 represents the gene expression pattern for granzyme B, CD3, CD25, CD152 and CD45 in the *ret*/PTC-1 positive cell line (TPC-1). As previously stated, figure 2 demonstrates the over-expression of these targets in the normal thyroid cell line model when exposed to H.T. lymphocyte supernatant in comparison to stimulation with control supernatant. However figure 5 illustrates that when a *ret*/PTC-1 positive thyroid cell line model is co-cultured with H.T. supernatant; in comparison to co-culture with control supernatant, this group of targets are under-expressed. This illustrates an important gene expression pattern, one that demonstrates a shift in expression of the cohort of targets when *ret*/PTC-1 activation is introduced.

It is noteworthy that, in figures 2 and 5, the cell line models that were not exposed to stimulus i.e. Nthy-ori at 6 h and TPC-1 at 6 h did not show any level of gene expression for the targets displayed. Relative quantification (RQ) values representing all co-culture combinations/time points for each assay have been normalized to a house keeping gene (Glyceraldehyde-3-phosphate dehydrogenase (GAPDH)) and then normalized to a calibrator sample (un-stimulated N-thy-ori or TPC-1 at 0 hours) according to the 2^-ΔΔC^t comparative threshold cycle (C_t_) method [5]. From this we can interpret that the gene expression patterns detected are a result of stimulation with H.T. or control lymphocytic supernatant and also the presence or absence of the *ret*/PTC-1 oncogene.

## Discussion

Although the pathogenic mechanisms determining thyrocyte destruction in Hashimoto Thyroiditis have been extensively investigated, it is still unclear exactly how this destructive process occurs.

Among oncogenes studied in the setting of thyroid neoplasia, activated c-*ret *-and *ret*/PTC-1 in particular has been found to associate with papillary thyroid carcinoma (PTC) and Hashimoto Thyroiditis (H.T.). Its presence has also been noted in lesions which have an affiliated inflammatory infiltrate. However, despite extensive studies into the prevalence and sequelae of the presence of activated *ret*/PTC-1 it remains unclear if *ret*/PTC-1 activation orchestrates, maintains or modulates the inflammatory response so often observed as a feature of *ret*/PTC-1 positive lesions.

The aim of this project was to observe the immune response elicited by a normal thyroid cell line model upon exposure to lymphocytic supernatant derived from a H.T. and normal donor at varying time points. To further evaluate the role of the *ret*/PTC-1 oncogene in a similar setting, we also observed the immune response elicited by an endogenously expressing *ret*/PTC-1 thyroid cell line stimulated with H.T. and normal lymphocyte supernatant. We chose to use an endogenously expressing *ret*/PTC-1 cell line rather than transfecting the normal cell line with *ret *as we felt that in the setting of this experiment, it was more biologically plausible to compare a normal thyroid cell line with a thyroid cancer cell line which natively expresses the oncogene. We decided to utilise supernatant from activated T-lymphocytes in each group to stimulate the cell line models to ensure assessment of gene expression accurately reflected the repertoire of the epithelial cells alone.

### Nthy-ori gene expression pattern

When thyroid cells from the Nthy-ori cell line model were exposed to Hashimoto Thyroiditis (H.T.) lymphocyte supernatant, a universal up-regulation of all immune targets was observed when compared to exposure to control lymphocyte supernatant (with the exception of Heme oxygenase-1 where expression between each cohort was equivocal) (figure 1).

However, within the global increased expression on stimulation by H.T. supernatant, some clusters of genes appeared to be particularly interesting. They included subsets of targets which may contribute to the pathogenesis of H.T. via cell mediated cytotoxic cell death and TCR signalling.

Thyrocyte specific CD8 cytotoxic T cell responses are a pivotal factor in the tissue specific destruction that is seen in H.T. Cell mediated cytoxicity is initiated through two different mechanisms 1) effector cell expression of death ligands presented to target cells and 2) effector cell release of exocytic granules containing perforin and granzyme B [6].

Over expression of both Fas ligand (FasL) and granzyme B, was seen when the normal thyroid cell line model was stimulated with H.T. lymphocyte supernatant compared to control supernatant.

Granzyme B is a serine protease that plays an important role in inducing apoptotic changes in target cells during granule exocytosis-induced cytotoxicity [7] while FasL induces apoptosis by binding to and activating its receptors, which in turn bind to adaptor molecules that activate the caspase family.

Increased expression of these cytotoxic agents in the normal thyroid cell line model exposed to H.T. lymphocyte supernatant suggests that these targets are specifically involved in cytotoxic cell death in H.T. leading to apoptosis of thyroid follicular epithelial cells and the resultant destruction of thyroid tissue that is indicative of H.T.

Moreover, targets involved with the T cell receptor (TCR) and T cell signalling were also up-regulated which may be responsible for exacerbation of the destructive process seen in H.T. These targets included CD3, CD25, CD28, CD152 and CD45.

T cell activation requires not only the generation of an initial signal through interaction of the antigenic peptide with the TCR-CD3 complex but also a subsequent antigen non-specific co-stimulatory signal, provided primarily by the interaction of CD28 on T cells with B7 molecules [8]. CD152 is implicated in this process via competitive binding to B7. CD45 is involved in maintaining protein tyrosine kinases in a dephosphorylated state prior to participation in signal transduction upon subsequent TCR ligation [9]. It is interesting to hypothesize that these over-expressed TCR and T cell signalling associated targets may be involved in the disease pathogenesis seen in H.T. via dysregulation of T cell signalling and control of T cell activation resulting in the over-activity of immune responses typically seen in autoimmune diseases such as H.T.

Finally, a large fold change difference (RQ value) was observed for granulocyte colony stimulating factor (G-CSF) when comparing thyrocytes exposed to H.T. and control lymphocyte supernatant in the normal cell line model (figure 3c). G-CSF elicits an effect on the immune system via its role in monocyte/macrophage activation, neutrophil stimulation and regulation of inflammatory cytokine/chemokine production. One cause of tissue damage in the thyroid is infiltration by lymphocytes and monocytes/macrophages. The over-expression of G-CSF in thyrocytes exposed to H.T. lymphocyte supernatant suggests that G-CSF may play a role in promoting the inflammatory aggravation and subsequent tissue destruction seen in autoimmune thyroid diseases such as H.T.

### TPC-1 gene expression pattern

We further wanted to evaluate the expression and role of the *ret*/PTC-1 oncogene in a similar context and so we reapplied our co-culture conditions to another thyroid cell line expressing this oncogene (TPC-1 cell line).

As previously stated, the Nthy-ori co-culture displayed up-regulation of a discrete subset of immune targets which may contribute to the disease pathogenesis seen in H.T. These targets included granzyme B, CD3, CD25, CD152 and CD45; targets which are involved in apoptosis, T cell signalling and T cell activation. In the *ret*/PTC-1 cell line model, these 5 targets showed a shift in gene expression pattern whereby they were down-regulated in *ret*/PTC-1 positive thyrocytes exposed to H.T. supernatant when compared to exposure to control supernatant. There has been much speculation surrounding the role that *ret*/PTC-1 plays in the inflammatory response in *ret *positive lesions. In this study we have seen that exposure to supernatant derived from H.T. peripheral blood mononuclear cells (PBMCs) results in the normal thyroid cell line model and the *ret*/PTC-1 positive thyroid cell line model producing different immunogenic patterns. Perhaps the expression of the *ret*/PTC-1 oncogene in the TPC-1 cell line acts to down-regulate apoptotic and T cell signalling targets, resulting in this change in gene expression pattern that we have seen. As a result of this expression pattern in the stimulated *ret*/PTC-1 expressing cell line, it is interesting to speculate that *ret*/PTC-1 activation in AITD may dampen the immune response and facilitate tumour growth and development.

*ret*/PTC has been shown to function in the MAPK (mitogen-activated protein kinase) signalling cascade by binding adaptor proteins such as growth receptor-bound protein 2 (GRB2), which in turn activate RAS. RAS activates AF kinases such as BRAF and its downstream signalling cascade including MEK (MAP kinase) and ERK (extracellular signal-regulated kinases or MAPK). Activated ERK1 and ERK2 translocate to the nucleus where they activate transcription factors such as ELK-1 and STAT3. Hwang and colleagues [10] have demonstrated that *ret*/PTC associates with and activates STAT3, while microarray analysis has demonstrated up-regulation of STAT3 in *ret*/PTC-3 thyrocytes [11]. Furthermore STAT3 has been shown to contribute to malignancy by inactivating intrinsic and extrinsic apoptotic pathways [12]. *ret*/PTC activation and the resultant STAT3 transcription factor activation via the MAPK signalling cascade may be involved in impeding apoptosis in thyrocytes by down-regulation of granzyme B. Although a link between STAT3 activation and dysregulation of apoptotic mechanisms via granzyme B down-regulation cannot be proven from this study alone it is interesting to speculate that STAT3 activation through *ret*/PTC MAPK signalling may contribute to malignancy in the setting of thyroid neoplasia.

## Conclusion

This study has highlighted a set of immune related gene expression markers which were shown to be up-regulated in a normal thyroid cell line model upon exposure to H.T lymphocyte supernatant when compared to control. These targets included genes which could plausibly be in involved in exacerbating the disease pathogenesis seen in H.T. To further ascertain the extent to which the oncogene *ret*/PTC-1 is involved in immunogenic responses in AITD, we used a *ret*/PTC-1 positive thyroid cell line model employing the same co-culture methods. We found that when this cell line was stimulated with H.T. supernatant, a shift in the immunogenic pattern was seen, whereby the same set of immune markers were down-regulated compared to control supernatant stimulation. We hypothesise that *ret*/PTC-1 expression in the TPC-1 thyroid cell line may lead to down-regulation of these immune targets. Consequently we can speculate that *ret*/PTC-1 activation in the setting of AITD may lead to under-expression of these immune targets, resulting in tumour growth and development. Nonetheless, this study, utilising TaqMan^® ^low density immune arrays, has given us further insight into the molecular immunogenesis of H.T. and some of the targets that may be involved in exacerbating disease pathogenesis.

## Methods

### Cell culture

Nthy-ori 3-1 (ECACC, Wiltshire, UK) represents a normal human thyroid follicular epithelial cell line which has been immortalised by transfection with a plasmid encoding for the SV40 large T gene.

TPC-1 is a cell line derived from a papillary thyroid carcinoma and expresses the *ret*/PTC-1 oncogene.

Both cell lines were grown to confluence in a humidified atmosphere containing 5% CO2 at 37°C in the following plating medium: RPMI 1640 with 2 m M L-glutamine (Invitrogen-GIBCO, MD, USA), 10% foetal calf serum (FCS) (Invitrogen-GIBCO, MD, USA), penicillin (100 U/ml) (Invitrogen-GIBCO, MD, USA) and streptomycin (100 U/ml) (Invitrogen-GIBCO, MD, USA).

### Lymphocyte isolation

Gene expression analysis was performed at three distinct time points for each cell type and co-culture variable; 0 hours, 6 hours and 18 hours. For each time point 12 mls of fresh whole blood was withdrawn from the H.T. and control subjects (lymphocytes were obtained from the same H.T. and control donor for all experiments) into heparinised Vacuette^® ^collection tubes (Greiner bio-one, Austria). Ethics approval was obtained from the Trinity College Faculty of Health Sciences Research Ethics Committee. Heparainised whole blood was treated with RosetteSep^® ^Human T cell Enrichment Cocktail (StemCell Technologies, UK), and the manufacturers' protocol for isolation of lymphocytes was followed. Briefly, fresh blood was incubated with RosetteSep^® ^cocktail (50 μl/ml of whole blood) at room temperature for 20 minutes. Samples were diluted with an equal volume of phosphate buffered saline (PBS) (Sigma-aldrich, MO, USA) and 2% FCS. Diluted samples were layered on top of density medium (Lymphoprep™, AXIS-SHIELD, Norway). Care was taken not to mix sample and density medium prior to centrifugation. Gradient centrifugation was performed at 4°C for 20 minutes at 1400 rpm (Eppendorf AG, 5810R, Germany). Enriched cells were removed from the density medium: plasma interface and washed with PBS and 2% FCS. T cells were pelleted, resuspended and counted using a haemocytometer. Approximately 2 × 10^6 ^T cells were isolated from 12 mls of whole blood. For each subject, a 24 well plate was plated with 1 × 10^6 ^T cells in two wells. In one of these wells, phorbol 12-myristate 13-acetate (PMA) (Sigma-Aldrich, MO, USA) (25 ng/ml) was added to activate the T cells. The second untreated well served as the control.

### Co-culture

After 3 days, the activated T cells were spun down and supernatant collected was added to wells plated with 2.5 × 10^5 ^Nthy-ori cells and 2.5 × 10^5 ^TPC-1 cells for the co-culture procedure. Table 1 shows the time points at which RNA was extracted from the thyrocytes and the various co-culture combinations.

**Table 1:** Time points at which RNA was extracted from thyrocytes and the various co-culture combinations

**0 Hour Time point**	**6 Hour Time point**	**18 Hour Time point**
Nthy-ori cells	Nthy-ori cells	Nthy-ori
TPC-1 cells	TPC-1 cells	TPC-1
	Nthy-ori cells + H.T lymphocyte supernatant	Nthy-ori cells + H.T lymphocyte supernatant
	TPC-1 cells + H.T lymphocyte supernatant	TPC-1 cells + H.T lymphocyte supernatant
	Nthy-ori cells + control lymphocyte supernatant	Nthy-ori cells + control lymphocyte supernatant
	TPC-1 cells + control lymphocyte supernatant	TPC-1 + control lymphocyte supernatant

Preliminary work (data not shown) co-culturing both thyroid cell lines with activated lymphocytes for time intervals of 6 and 18 hours resulted in strong aggregation of thyrocytes and lymphocytes impairing isolation of pure populations of thyrocytes for gene expression analysis. This phenomenon was most likely due to HLA incompatibility and/or contact inhibition. Hence, it was decided to extract supernatant from activated T-cells for stimulation of thyrocytes to provide a uniform trigger across both *ret*/PTC-1 + and - cell types.

### RNA extraction and cDNA archiving

At 0, 6 and 18 hour time points' cells were tripsinised (Sigma-aldrich, MO, USA) and centrifuged to remove supernatant. RNA was extracted using RNeasy ^® ^mini kit (Qiagen Ltd., West Sussex, UK) according to manufacturer's protocol. RNA quantity was assessed using a NanoDrop^® ^ND-1000 Spectrophotometer (Wilmington, USA). The RNA was reverse transcribed to single stranded cDNA using a High Capacity cDNA Archive Kit (Applied Biosystems, CA, USA) in 100 μl reactions.

Reactions contained 10 μl of buffer (10×), 4 μl of deoxynucleotide triphosphate (25×), 10 μl of random primers (10×), 5 μl of multiscribe RT enzyme (50 U/μl), 21 μl of nuclease-free water and 50 μl of extracted total RNA (20 ng/μl).

The reactions were incubated at 25°C for 10 minutes and 37°C for 2 hours (Perkin Elmer 9600 GeneAmp PCR system, Applied Biosystems, CA, USA).

### TaqMan^® ^low density array (TLDA)

Gene expression analysis was performed using TaqMan^® ^immune-profiling/inflammation low density arrays (TLDAs). The panel of assays included in the TLDA format is designed to simultaneously assess gene expression patterns of multiple targets in a micro-fluidic system. Each immune profiling TLDA contains lyophilised gene expression reagents (primers and probes [FAM labeled]) in a preconfigured 384 well format (see additional file 1 for an illustration of the gene expression reagents that were factory loaded onto the TaqMan^® ^TLDAs). Each of the 8 separate loading ports feeds into 48 separate wells, with 4 well replicates per assay.

One sample was analysed per card. Each loading port was filled with a 100 μl volume containing 5 μl cDNA (20 ng/μl), 45 μl of nuclease free water and 50 μl of TaqMan^® ^universal PCR master mix (2×). After loading, the cards were placed in Sorvall^®^/Heraeus^® ^custom buckets (Applied Biosystems, CA, USA) and centrifuged in a Sorvall Legend T EASYset centrifuge (Thermo Electron Corporation, Germany) for a total of 2 consecutive 1 minute spins at 1200 rpm to ensure complete distribution of the sample from the loading port into each well. Following centrifugation, cards were sealed with a TLDA sealer (Applied Biosystems, CA, USA) to prevent cross contamination. Real time RT-PCR amplification was performed on an ABI Prism^® ^7900 H.T. Sequence Detection system (Applied Biosystems, CA, USA). Thermal cycling conditions were as follows: 2 minutes at 50°C, 10 minutes at 94.5°C, and for 40 cycles, 30 seconds at 97°C and 1 minute at 59.7°C.

### TaqMan^® ^low density array analysis

TLDA cards were analyzed with relative quantification (RQ) documents and RQ manager software for automated data analysis (Applied Biosystems, CA, USA). For analysis purposes, the Nthy-ori time points and the TPC-1 time points were analysed as two separate RQ studies. Expression values were calculated using the comparative threshold cycle (C_t_) method. Briefly, this technique uses the formula 2^-ΔΔC^t [5] to calculate the expression of target genes normalised to a calibrator (un-stimulated N-thy-ori or TPC-1 at 0 hours). Glyceraldehyde-3-phosphate dehydrogenase (GAPDH) was selected as the endogenous control.

The threshold cycle C_t _indicates the cycle number by which the amount of amplified target reaches a fixed threshold. The C_t _data for all inflammation target genes and GAPDH were used to create ΔC_t _values [ΔC_t _= C_t _(target gene)-C_t _(GAPDH)]. ΔΔC_t _values were calculated by subtracting the calibrator (Nthy-ori at 0 hrs or TPC-1 at 0 hrs) from the ΔC_t _value of each target. Relative quantification (RQ) values were calculated using the equation 2^-ΔΔC ^_t._

## Competing interests

The authors declare that they have no competing interests.

## Authors' contributions

KD carried out the co-culture and gene expression analysis and wrote original and final versions of this manuscript.,PS carried out statistical and analytical analysis of gene expression data and helped draft the manuscript, SC helped with analysis of gene expression data and carried out cell culture, RF helped with analysis of gene expression data, JHL and SA carried out cell culture, JOL and OS conceived the study and helped write the manuscript. All authors have read and approved the final manuscript.

## Supplementary Material

Additional file 1 Table 2. (courtesy of Applied Biosystems) illustrates the gene expression reagents (primers and probes) that were factory loaded onto the TaqMan^® ^immune profiling low-density arrays. Click here for file
