# Supplementary figures and images for: ret/PTC-1 expression alters the immunoprofile of thyroid follicular cells
Source: Mol Cancer. 2008 May 27;7:44. doi: 10.1186/1476-4598-7-44 (PMC2423371; doi:10.1186/1476-4598-7-44)

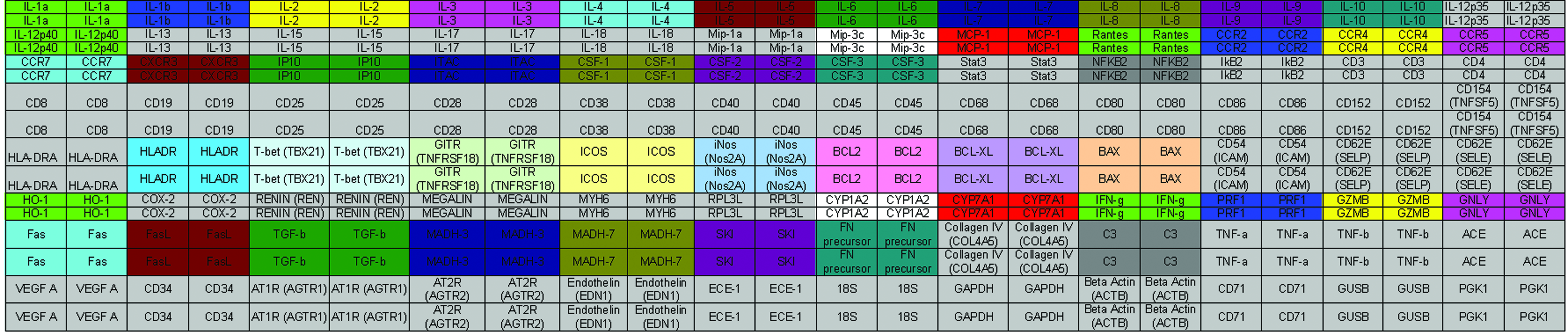

Supplement: Additional file 1 — Table 2. (courtesy of Applied Biosystems) illustrates the gene expression reagents (primers and probes) that were factory loaded onto the TaqMan® immune profiling low-density arrays. [file 1476-4598-7-44-S1.tiff]
